# The Psychosocial Impact of the COVID-19 Pandemic on Italian Families: The Perception of Quality of Life and Screening of Psychological Symptoms

**DOI:** 10.3390/pediatric16020043

**Published:** 2024-06-20

**Authors:** Roberta Maria Incardona, Marta Tremolada

**Affiliations:** Department of Development and Social Psychology, University of Padua, Via Venezia 8, 35131 Padova, Italy; robertamaria.incardona@phd.unipd.it

**Keywords:** COVID-19, quality of life, psychological symptoms, children, adolescents, parents, predictive models

## Abstract

Throughout the COVID-19 period, families were forced to stay indoors, adapting to online schooling, remote work, and virtual social engagements, inevitably altering the dynamics within households. There was a notable increase in mental health challenges in terms of anxiety and depression in children and adolescents. This study intended to explore the psychosocial effects of the COVID-19 pandemic on Italian families by adopting self- and proxy-report questionnaires on anxiety, anger, and health-related quality of life. The results showed that approximately 20% obtained a clinical anxiety score and only 10% obtained a clinical anger score. There was a difference in the perception of the quality of life reported by the child and that perceived by the parent. A stepwise regression model showed that total anxiety scores were predicted by sex, quality of life scores from the parents’ self-report version, and the total anger score. Another stepwise regression model identified physiological and social anxiety as the best predictors that impact quality of life. Parental well-being actively influences the well-being of children, so it is fundamental to implement preventive programs and promote child well-being by providing parents the most adequate support possible.

## 1. Introduction

The onset of the COVID-19 pandemic brought about an era of unparalleled global upheaval, significantly affecting people of various demographic backgrounds. Throughout this period, families found themselves forced to stay indoors, adapting to online schooling, remote work, and virtual social activities, inevitably altering the dynamics within households [1,2]. Parents were forced to simultaneously manage their professional responsibilities, childcare activities, and housework, leading to increased stress, fatigue, and consequently negatively affecting their productivity [1,3,4,5].

Furthermore, the period of social isolation introduced various stressors and concerns that significantly affected the well-being and mental health of children and adolescents. Abrupt disruptions in daily routines, including sudden interruptions in school attendance and limited contact with peers and relatives, together with hidden or manifest concerns of parents, contributed to increased uncertainty [6]. As is known, psychological development is characterized by transformations and challenges as individuals strive for autonomy from their parents, construct their identity [7,8], and, particularly during adolescence, undergo social development and a greater need for social interactions [9]. 

In this regard, the findings of cross-sectional and longitudinal investigations, as well as systematic reviews and meta-analyses on the psychological impact of the COVID-19 lockdown and the subsequent periods, highlight a marked decline in health-related quality of life (HRQoL) among children and adolescents [7,10,11]. Additionally, there were psychological consequences for all family members, including increased levels of anxiety, depression, and sleep problems [12]. 

Therefore, it can be stated that the COVID-19 pandemic worsened the quality of life and well-being of many people, particularly those who lived in a situation of physical, psychological, or social hardship even before the outbreak of the health emergency [13]. Families with school-age children were most affected because they had to give them support and take care of their development in stressful, difficult conditions [5].

Regarding the HRQoL assessment, it is important to note that the literature shows that there is a widely acknowledged gap between children’s self-reported information and that provided by their parents regarding the children’s health and well-being [13,14]. This incongruence is extensively documented in the pediatric and adult literature, and proxy assessors often underestimate the quality of life related to child health compared to self-reports [15,16,17]. This divergence can be attributed to various factors related to the child, the parent, and the specific domains of quality of life related to health being considered [14]. In child populations, proxy HRQoL assessments can be influenced by external variables, such as a parent’s own HRQoL [18]. While parental input is valuable when children cannot provide independent responses, it is crucial to recognize that children’s self-reports offer a more accurate reflection of their health status. Ideally, individuals themselves should serve as primary informants about their HRQoL, symptoms, sensations, and health conditions [14,19].

At the same time, in this post-COVID-19 era, there has also been a notable increase in mental health challenges in terms of anxiety and depression in children and adolescents [10,11,20]. De Bles et al. [21] suggest a possible association between anxiety and depressive disorders and possible links of the last two also with trait anger, even if it depends on the definition of the construct of anger [22].

Regarding psychopathology, several studies indicated an increase of 83% in the number of accesses to child neuropsychiatry services during the pandemic period [23], and anxiety has become the most prevalent mental disorder among the youth population in Europe [24]. Both fatigue and parental stress are identified as risk factors for the development of negative mental health outcomes in both parents and children [25]. Moreover, this is related to parents’ perception of children’s executive functions (EFs): the most distressed parents perceived their children as less competent in EF, highlighting a cognitive fragility in attention, memory, and self-regulation [26]. Spinelli et al. [5] found that parental perception, particularly their struggles in managing the various stressors imposed by quarantine, was significantly linked to parental stress and psychological problems in children. 

Furthermore, another investigation [27] highlights the significant impact of parental involvement (PI) and parental stress on children’s academic adjustment and overall quality of life (QoL).

### Aims

The final objective of this study is to understand the associations between the impact of COVID-19, mental health symptoms, and the quality of life of children and adolescents and their parents.

Specifically, the following study intends to perform the following:

1. Conduct a screening of anxiety, anger symptoms, and quality of life (QoL) in Italian children, adolescents, and their parents using self- and proxy-report tools based on data available in the literature.

2. Investigate the disparities between children’s self-reported perception of quality of life and the perception reported by their parents (proxy-report), with a specific focus on evaluating the role of fatigue in children’s quality of life.

3. Understand the associations between various symptoms, sociodemographic variables, and parental well-being by proposing predictive models on anxiety and quality of life.

## 2. Materials and Methods

### 2.1. Participants

The first sample consisted of 131 subjects aged between 6 and 16 years; the average age was 12.44 years (SD = 2.743; range = 6–16 years). The sample was characterized by 60 boys (45.8% of the total) and 71 girls (54.2% of the total). The descriptive statistics regarding the attended class indicated that 35 subjects attended primary school (26.7% of the total), 50 subjects attended lower secondary school (38.2% of the total), and 46 subjects attended upper secondary school (35.1% of the total).

The second sample consisted of 131 subjects aged 30 to 66 years, with an average age of 46.46 years (SD = 6.025; range = 30–66 years). Only one subject did not specify their age. The descriptive statistics and frequencies regarding the data collected from the sociodemographic questionnaire administered to the subjects and their parents are summarized in Table 1.

### 2.2. Procedure

After obtaining consent from the Psychology Ethics Committee (protocol code 4039, University of Padua), both parents of the children signed the written informed consent form. During this process, the study’s objectives, the necessary information for the research, data collection methods, and the required duration for data collection were specified. After completing the informed consent form, the families received two links to access the questionnaires anonymously and securely on the LimeSurvey platform—one link was reserved exclusively for the child and one for the parent. It was specified that for each child, only one parent would complete the assigned questionnaires. The data necessary for this study were collected between April and June 2022 through personal meetings with psychology students at the University of Padua in different regions of the country. To ensure anonymity and prevent the identification of participants, specific data about their region of origin were not collected; only their affiliation with the Veneto region or otherwise was requested.

### 2.3. Instruments

#### 2.3.1. Sociodemographic Questionnaire

Parents filled out a sociodemographic questionnaire that included questions about themselves, including their sex, highest year of schooling, education, perceived economic situation, type of home situation, romantic relationship status, and type of employment.

#### 2.3.2. Revised Children’s Manifest Anxiety Scale—Second Edition 

The Revised Children’s Manifest Anxiety Scale [28] is a brief self-report inventory measuring the level and nature of anxiety in 6- to 19-year-olds. Designed as a self-administered instrument using “yes” and “no” questions, this instrument has been validated and found reliable for use in children, with norms reported in 3 age groups: 6–18, 9–14, and 15–19 years. The test is now composed of 49 items that cover the following scales: physiological anxiety (PHY), worry (WOR), social anxiety (SOC), defensiveness (DEF), inconsistent responding index (INC), and total anxiety (TOT). Reliability of the scales and subscales of the RCMAS-2 is reported via the Cronbach alpha as follows: 0.92 for TOT, 0.75 for PHY, 0.86 for WOR, and 0.80 for SOC [28]. The Cronbach alpha estimate for the RCMAS-2 represents the homogeneity of the instrument items and scale scores and is adequate in reliability. The validity of the RCMAS-2 was established via interscale correlations with moderate to high ranging from 0.59 to 0.73 for the anxiety scales and 0.83 to 0.93 for the scales and TOT [29].

Scoring was conducted exclusively through the Giunti Psychometrics online testing platform, Giunti Testing, regardless of the administration method. The scoring process was automatic, calculating the subject’s score and producing a graphic profile. The scores on the content scales were standardized and referred to as normalized T-scores: they have a mean of 50 and a standard deviation of 10. The term ‘normalized’ indicates that each value of a T-score corresponds to the same percentile rank across all scales.

#### 2.3.3. ChIA 

The Children’s Inventory of Anger (ChIA) [30] is a self-administered questionnaire designed to identify situations that provoke anger in children and adolescents, as well as the intensity of their responses to these situations. The ChIA is short, quick, and cost-effective, making it one of the few tools that provide insight into the child’s and adolescent’s perspective on their anger. It consists of 39 items and provides a total score, an Incongruent Response Index (IRI), and scores for four subscales: frustration, physical aggression, relationships with peers, and relationships with authorities. The statements are written in a simple manner to ensure comprehension by young children or those with learning difficulties, yet are sophisticated enough to be accepted by older children without being perceived as childish. The answer options in the ChIA correspond to four pictograms displaying expressions ranging from happiness to extreme anger. The respondent marks the pictogram that best represents how angry they would feel in the described situation. 

Scoring was conducted exclusively through the Giunti Psychometrics online testing platform, Giunti Testing, regardless of the administration method. This process was automatic and, besides calculating the score, it also generated a graphical report. ChIA scores were transformed into normalized T scores, which have a mean of 50 and a standard deviation of 10. ‘Normalized’ indicates that each T score value on each scale corresponds to the same percentile rank as the T score value on the other scales. Higher scores reflect higher levels of anger, while lower scores indicate lower levels of anger. The ChIA’s internal consistency reliability was evaluated using coefficient alpha. In a study involving 1604 young participants, impressive values were obtained, with coefficients of 0.95 for the total scale and 0.85–0.86 for each of the four subscales. Additionally, test–retest reliability was assessed in a sample of 87 children aged 6–11 over a 1-week interval, resulting in a coefficient of 0.75 for the total scale and ranging from 0.65 to 0.75 for the subscales. Furthermore, content validity for the instruments was deemed satisfactory based on feedback from professionals who used the scale, while concurrent validity was established through the examination of the ChIA’s relationship with other measures [30].

#### 2.3.4. PedsQoL 3.0 Multidimensional Fatigue Scale 

The PedsQoL 3.0 Multidimensional Fatigue Scale [31] is an 18-item questionnaire designed to assess three dimensions of fatigue: general fatigue, sleep-related fatigue, and cognitive fatigue in individuals aged 2 years and older. The scale aims to determine how fatigue impacts health-related quality of life (HRQOL). It includes a self-report version for children aged 5 years and older, and a proxy-report version for parents for children aged 2 years and older. This tool is simple and quick to administer, either individually or in groups, and can be conducted using paper and pencil or as a semi-structured interview. Responses are given on a five-level Likert scale, where 0 corresponds to “Never” and 4 to “Almost always”, indicating the frequency with which a subject perceives or identifies with the sensations and situations described by the items. 

The scoring process was conducted manually, following the guidelines outlined in the manual. The first step involved transforming the scores, whereby each item was classified with an inverse score and then linearly transformed within a scale of values ranging from 0 to 100. The transformation of response values is as follows: 0 = 100; 1 = 75; 2 = 50; 3 = 25; 4 = 0. For each dimension (general fatigue, sleep-related fatigue, and cognitive fatigue), the score was derived by calculating the average value of the transformed scores of the six items comprising it. Similarly, the total score was obtained by averaging the transformed scores of all 18 items in the questionnaire. According to the scoring manual, higher scores are associated with better health-related quality of life and fewer problems or symptoms, whereas lower scores indicate greater fatigue and poorer quality of life. The PedsQL Multidimensional Fatigue Scale Total and Subscale internal consistency reliabilities demonstrated an alpha coefficient standard of 0.70 for group comparisons for child self-report ages 5–18 years and parent proxy-report ages 2–18 years. Across the ages, it approached or exceeded an alpha of 0.90, recommended for individual patient analysis, making the PedsQL Multidimensional Fatigue Scale Total Score suitable as a summary score for the primary analysis of HRQOL fatigue outcome in clinical trials and other group comparisons. The General Fatigue, Sleep/Rest Fatigue, and Cognitive Fatigue Subscales may be utilized to examine specific dimensions of fatigue and are recommended for secondary analyses.

### 2.4. Plan of Statistical Analyses

Descriptive analyses determined whether anxiety and anger levels in children and adolescents fall within clinically significant ranges or within normal distribution, based on specific cut-off points. Following this, correlational analyses were performed, followed by stepwise regression models, to elucidate the factors influencing the quality of life in children and adolescents following the COVID-19 period.

## 3. Results 

### 3.1. Perceptions of Anxiety, Anger, and Quality of Life of Children and Adolescents Compared to Norms

**Figure 1:**
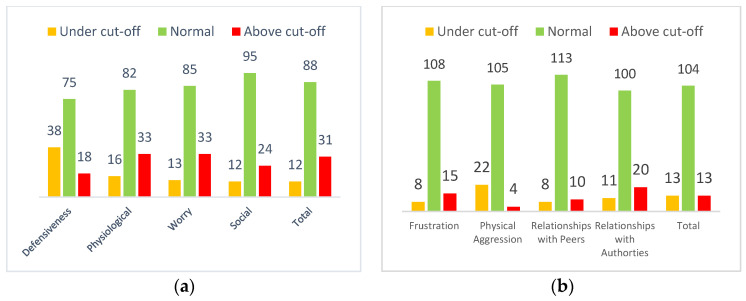
Distribution of children’s and adolescents’ scores on the anxiety scales of the RCMAS-2 test (**a**) and on the ChIA scores (**b**).

The RCMAS-2 test scores were divided into the scales described and identified in the Reynolds and Richmond manual [28]. The results showed that approximately 70% of the sample obtained an average total anxiety score, while approximately 20% obtained an above-average anxiety score (Figure 1a). 

Regarding the first objective of this research, approximately 80% of the sample obtained an average test score in the ChIA. Only 10% obtained a score above the clinically significant average; this suggests that the distribution of anger levels in the present sample is on average normal and not clinically significant (Figure 1b).

The scores of the PedsQLTM 3.0 Multidimensional Fatigue Scale test were converted according to the manual instructions and were recorded and divided into four categories that qualitatively describe the score in relation to the subject’s quality of life (Table 2).

Most of the children and adolescents reported a moderate total quality of life (62.72%), while 10.7% scored a low quality of life and 22.1% a high quality of life. 

Parents also self-reported QoL scores, showing a worse picture with 19.1% reporting low perception (Table 3). 

### 3.2. Comparison of Quality of Life Scores between Parents and Children

**Figure 2:**
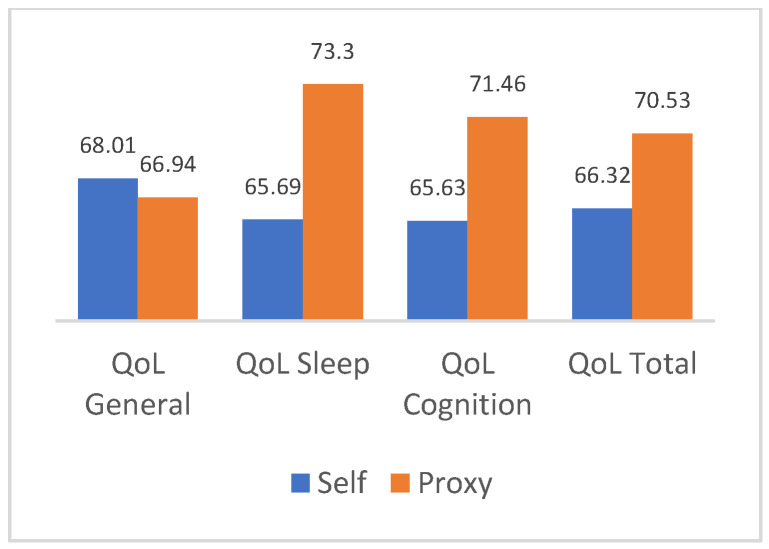
Comparison of self-report and proxy means of quality of life scores.

A paired-sample *T*-test was performed to compare the mean scores of the various areas of quality of life reported by children and adolescents with those reported by parents. Significant differences (*p* < 0.005) were found for the sleep area (t = −4.61, df = 130), the cognitive area (t = −2.91, df = 130), and the overall quality of life (t = −3.09, df = 130). Thus, the second objective of the study, which aimed to examine differences in the perception of quality of life between children and their parents, was confirmed. The results of the analysis are reported in Figure 2. 

### 3.3. What Are the Factors That Influence Anxiety Symptoms in Children and Adolescents?

First, a series of bivariate Pearson correlations were conducted among the sociodemographic variables of the children, parental quality of life, anger scores, and anxiety symptoms. Based on these correlations, hierarchical linear regression models were proposed using the stepwise method.

In the first linear regression, the total anxiety scores on the RCMAS-2 test were set as the dependent variable, with sex, quality of life scores from the parents’ self-report version, and total anger scores on the ChIA test as independent variables. The third model was found to be significant (*p* < 0.005) and explains most of the variance (15%). The mentioned variables emerged as significant predictors of the dependent variable (See Table 4).

### 3.4. What Are the Factors That Influence the Perceived Quality of Life of Children and Adolescents?

The second proposed hierarchical linear regression established the scores of total quality of life from the self-report version provided by the children/young people as the dependent variable. The independent variables included the scores of physiological anxiety and social anxiety, which are anxiety subscales of the RCMAS-2 test. This model was found to be significant (*p* < 0.005) and explained a substantial portion of the variance (33%). The aforementioned variables emerged as significant predictors of the dependent variable (See Table 5).

## 4. Discussion

The primary objective of this study was to conduct a screening of anxiety-related disorders, anger levels, and the overall quality of life among Italian children and adolescents. The findings indicate that more than half of the participants scored within the age-specific average range. However, concerning the total anxiety scale and its physiological anxiety and worry subscales, over 20% of the sample obtained clinically relevant scores, exceeding the average for their age group. For the social anxiety subscale, only 18.3% of the sample achieved clinically significant scores above the age-specific average. These outcomes underscore the prevalence of anxiety disorders in the child population and corroborate the findings of Racine et al.‘s meta-analysis [11], which revealed that one in five children exhibited clinical symptoms of anxiety. Furthermore, comparing pre-pandemic data, Racine’s study suggests that mental health challenges among youth have doubled [11]. This study confirms the elevated prevalence of low HRQoL, mental health problems, and anxiety after the pandemic [6,7]. Probably, the decrease in adolescent face-to-face contact might be less detrimental due to widespread access to digital forms of social interaction through technologies such as social media [10].

Only 15% of the sample exhibited clinically significant levels of anger, particularly for the anger toward authority subscale. This observation may be attributed to the predominant composition of adolescents in the sample, wherein it is common for individuals to experience anger and frustration towards adult figures wielding authority over them. Moreover, the stringent restrictions imposed on children and adolescents during the COVID-19 pandemic likely exacerbated feelings of frustration, resulting in hostility towards authority figures [32]. Interestingly, a minimal proportion of subjects, approximately 3% of the sample, displayed clinically significant high levels of the physical aggression subscale, while roughly 17% exhibited extremely low levels of anger, both clinically significant. These findings contrast with those of Reid and colleagues’ study [32] which investigated psychological distress and the prevalence of antisocial behaviors among young people in the American population before and after the onset of the COVID-19 pandemic. Reid’s study noted a surge in frustration among subjects, consequently leading to increased aggressive behaviors. 

In this sample, the levels of physical aggression fell below the normal threshold for the age group, suggesting that anger may have been internalized and directed inward rather than outward, towards the self rather than others. While their anger levels appeared to be within the normal range, data regarding the subjects’ levels of physical aggression indicated that a significant portion of the sample exhibited aggression below the expected threshold. This suggests that aggression among the subjects in this sample may have been turned inward, contributing to the internalization of anger symptoms. Such internalization could have exacerbated the individual’s anxious internalizing symptoms and, consequently, negatively impacted psychological well-being.

The results indicated that children generally have relatively high levels of quality of life, with around 80% of the sample obtaining medium-high values across all areas. However, approximately 20% of the sample reported lower levels of quality of life in sleep-related and cognitive fatigue areas. Probably, children less competent in executive functions could have more cognitive fragility in attention, memory, and self-regulation [26], also in association with cognitive fatigue. Interestingly, there appeared to be a sustained decline in overall HRQoL throughout the COVID-19 pandemic that did not necessarily subside when the lockdowns ended [10]. 

As for the parents’ group, on average, 70% of the sample reported medium–high levels of quality of life across all areas. Notably, general and cognitive fatigue significantly compromised the quality of life levels, with 35% and 25% of the subjects reporting low quality of life levels in these areas, respectively. These findings align with previous studies [13,25] that have identified a decrease in health-related quality of life and an increase in anxiety and stress levels following the COVID-19 pandemic.

The second aim of the study was to explore any disparities in the perception of quality of life between children or adolescents and their parents, as assessed through self-report and proxy-report versions in assessing fatigue. Findings indicated discrepancies between children and parents in their perception of quality of life across all areas, especially concerning the assessment of overall quality of life. The data implied that parents may tend to overrate their children’s well-being. These findings corroborate the existing literature, highlighting significant variances between self-report and proxy-report versions in evaluating internal states and health status [14,15].

To address the third objective of the study, which aimed to identify potential factors influencing anxiety symptoms in children, various variables were examined. Gender, parental perception of quality of life, and children’s levels of total anger were found to significantly impact anxiety symptoms. These findings align with the existing literature. The association between female gender and higher anxiety levels supports previous research linking female sex with increased anxiety and depressive symptoms during childhood and adolescence [11]. Furthermore, the following study revealed that parental quality of life, particularly fatigue, predicts the mental health status of their children, specifically their anxiety symptomatology. The way parents manage stress, both individually and in their interactions as a dyad, plays a significant role in influencing their children’s emotional and behavioral well-being [5]. Additionally, the emotional support provided by parents serves as a crucial protective factor against the development of psychopathological symptoms in their children [6]. Contrarily, parental stress, burnout, depression, anxiety, and anger of parents toward children could be risk factors for their psychological health [2].

The final factor investigated highlighted how the levels of anger in children or adolescents can predict their anxiety levels, corroborating the existing literature. Studies on adults with anxiety disorders have consistently shown elevated rates and intensity of anger compared to control groups, with anger severity being closely linked to the severity of anxiety disorders [33]. Generally, higher levels of anger are frequently observed in individuals with depression or anxiety disorders [34].

Moreover, significant predictive factors related to the overall quality of life in children were identified, notably their levels of physiological and social anxiety. These results are in line with the existing literature on the impact of anxiety disorders on quality of life. This impact appears to be substantial, independent of symptom severity, demographic factors, somatic health, and diagnostic comorbidity [34]. The decline in quality of life, particularly in the social domain, among individuals with anxiety disorders could potentially contribute to the development of psychiatric comorbidities and exacerbate overall health conditions. Physical distancing measures to contain the spread of COVID-19 removed many sources of face-to-face social connection from people’s lives, which may have affected people’s mental health, particularly in adolescence, a period of life characterized by a heightened need for peer interaction [10]. Furthermore, low quality of life may pose a risk factor for relapse following the successful treatment of anxiety disorders [34]. 

Regarding the results in relation to the last objective of the present study, the resulting clinical implications were different. Firstly, it emerged that the individual’s quality of life is strongly influenced by the subject’s social anxiety and worries; additionally, it was observed that an individual’s total anxiety levels are influenced and predicted by the quality of life reported by their parents, as well as the individual’s own level of anger. 

Considering these findings, it is crucial to emphasize the importance of implementing anger management and control programs in initiatives aimed at prevention and promoting health and well-being. By addressing anger issues, overall well-being can be enhanced, and the escalation of anxious symptoms can be prevented. To promote mental health in children and adolescents effectively, it is essential to incorporate anger management interventions. Doing so can help minimize the emergence of anxious symptoms, thereby preventing a decline in the individual’s quality of life.

One of the study’s strengths concerns the sample size which was quite high and encompassed participants from various regions of Italy. The sample was notably homogeneous, and the children’s group spanned a broad age range. The utilization of various standardized tests with robust psychometric properties enabled a thorough analysis of associated constructs, facilitating a deeper understanding of their interrelationships. Another notable strength is the collection of information from parents, enabling an exploration of the relationship between their well-being and that of their children.

Possible limitations of the study include the absence of pre-pandemic mental health data for the subjects, hindering comparisons and insights into its impact. Additionally, the parent sample was heterogeneous, with a predominance of mothers. 

A final limitation is that the responses to the items of the various tests and questionnaires administered to the subjects may have been influenced by social desirability biases. Participants might have altered their responses due to concerns about presenting a negative or overly problematic image of themselves. Additionally, the small sample size is another limitation to consider.

These research perspectives could contribute to a better understanding of the long-term effects of COVID-19 on the mental health of young people and their families through longitudinal studies. The development and effectiveness of specific interventions for anger management in young people during stressful situations, such as those caused by the pandemic, could also be deepened. These interventions could be designed to improve emotion regulation skills and reduce internalized anger, with a focus on anxiety and psychological well-being. Additionally, studying the relationship between these variables and further information regarding the health status of parents could provide valuable insights into how different psychological issues in parents influence their children. Future research could also include data on specific clinical populations to understand how the COVID-19 pandemic has affected well-being and mental health across various groups.

## 5. Conclusions

To conclude, it is essential to pay attention to the mental health of children and their parents. The well-being of parents has a direct influence on that of their children; therefore, in prevention and well-being promotion programs for children, it is crucial to also consider the role of parents to provide them with adequate support to cope with stress and improve their own well-being. This study has also highlighted that anger is strongly correlated with anxiety, which in turn is linked to quality of life. Consequently, in prevention and well-being promotion programs, it is important to consider these interconnections and to organize comprehensive interventions that include the management of externalizing symptoms such as anger to prevent the onset of anxiety symptoms.

## Figures and Tables

**Table 1:** Sociodemographic characteristics of samples.

	Statistics					Frequencies	
		Min	Max	M	SD		
Children’s Age		6	16	12.44	2.74		
Children’s Gender	Male Female					60 71	45.8% 54.2%
Children’s School Level	Primary School Secondary School, 1st Secondary School, 2nd					35 50 46	26.7% 38.2% 35.1%
Parental Age		30	66	46.46	6.02		
Parent’s Gender	Male Female					26 105	19.8% 80.2%
Parental Schooling Years		5	20	13.82	3.42		
Parental Civil Status	Single Parent Two Parents					10 121	7.6% 92.4%
Parental Perceived Economic Condition	Low Medium High					23 72 36	16.6% 55.0% 27.5%

**Table 2:** Distribution of quality of life scores along with children’s and adolescents’ self-reports.

	QoL General		QoL Sleep		QoL Cognition		Total QoL Total	
	Freq.	Perc.	Freq.	Perc.	Freq.	Perc.	Freq.	Perc.
Very Low QOL	1	0.8%	6	4.6%	6	4.6%	0	0%
Low Quality of Life	21	16%	19	14.5%	21	16%	14	10.7%
Moderate QoL	72	55%	75	57.3%	65	49.6%	88	67.2%
Good QoL	37	28.2%	31	23.7%	39	29.8%	29	22.1%
Total	131	100%	131	100%	131	100%	131	100%

**Table 3:** Distribution of quality of life scores along with parental self-reports.

	QoL General		QoL Sleep		QoL Cognition QoL Total			
	Freq.	Perc.	Freq.	Perc.	Freq.	Perc.	Freq.	Perc.
Very Low QOL	8	6.1%	0	0%	4	3.1%	1	0.8%
Low QoL	36	27.5%	16	12.2%	30	22.9%	25	19.1%
Moderate QoL	66	50.4%	67	51.1%	50	38.2%	76	58%
Good QoL	21	16%	48	36.6%	47	35.9%	29	22.1%
Total	131	100%	131	100%	131	100%	131	100%

**Table 4:** Stepwise regression model with anxiety in children and adolescents as dependent variable.

	Model	Anova ^a^					Coefficients ^a^
	R-Square	Df	F	*p*	*Beta*	T	P
Model	0.15	3	7.6	0.0001 ^b^			
Sex					0.22	2.68	0.008 *
Parents of PEDS TOT Self-Report					−0.197	−2.40	0.018 *
Total Anger					0.24	2.93	0.004 *

^a^ Dependent variable: total anxiety. ^b^ Predictors: sex, PEDS TOT self-report parents, total anger. * p < 0.005.

**Table 5:** Stepwise regression model with self-report quality of life for children and adolescents as dependent variable.

	Model	Anova ^a^					Coefficients ^a^
	R-Square	Df	F	*p*	*Beta*	T	P
Model	0.33	2	31.67	0.0001 ^b^			
Physiological Anxiety					−0.34	−4.12	0.0001 *
Social Anxiety					−0.32	−3.90	0.0001 *

^a^ Dependent variable: PEDS TOT self-report children/adolescents. ^b^ Predictors: physical anxiety, social anxiety. * p < 0.005.

## Data Availability

The raw data supporting the conclusions of this article will be available from the authors on request.
